# Production and characterization of no-carrier-added ^161^Tb as an alternative to the clinically-applied ^177^Lu for radionuclide therapy

**DOI:** 10.1186/s41181-019-0063-6

**Published:** 2019-07-10

**Authors:** Nadezda Gracheva, Cristina Müller, Zeynep Talip, Stephan Heinitz, Ulli Köster, Jan Rijn Zeevaart, Alexander Vögele, Roger Schibli, Nicholas P. van der Meulen

**Affiliations:** 1 Center for Radiopharmaceutical Sciences ETH-PSI-USZ, Paul Scherrer Institute, 5232 Villigen-PSI, Switzerland; 2 Laboratory of Radiochemistry, Paul Scherrer Institute, 5232 Villigen-PSI, Switzerland; 3 Institut Laue-Langevin, 38042 Grenoble, France; 4 Radiochemistry, South African Nuclear Energy Corporation (Necsa), Brits, 0242 South Africa; 5 Department of Chemistry and Applied Biosciences, ETH Zurich, 8093 Zurich, Switzerland

**Keywords:** ^161^Tb, Auger/conversion electrons, Purification method, Somatostatin analogues, DOTA

## Abstract

**Background:**

^161^Tb is an interesting radionuclide for cancer treatment, showing similar decay characteristics and chemical behavior to clinically-employed ^177^Lu. The therapeutic effect of ^161^Tb, however, may be enhanced due to the co-emission of a larger number of conversion and Auger electrons as compared to ^177^Lu. The aim of this study was to produce ^161^Tb from enriched ^160^Gd targets in quantity and quality sufficient for first application in patients.

**Methods:**

No-carrier-added ^161^Tb was produced by neutron irradiation of enriched ^160^Gd targets at nuclear research reactors. The ^161^Tb purification method was developed with the use of cation exchange (Sykam resin) and extraction chromatography (LN3 resin), respectively. The resultant product (^161^TbCl_3_) was characterized and the ^161^Tb purity compared with commercial ^177^LuCl_3_. The purity of the final product (^161^TbCl_3_) was analyzed by means of γ-ray spectrometry (radionuclidic purity) and radio TLC (radiochemical purity). The radiolabeling yield of ^161^Tb-DOTA was assessed over a two-week period post processing in order to observe the quality change of the obtained ^161^Tb towards future clinical application. To understand how the possible drug products (peptides radiolabeled with ^161^Tb) vary with time, stability of the clinically-applied somatostatin analogue DOTATOC, radiolabeled with ^161^Tb, was investigated over a 24-h period. The radiolytic stability experiments were compared to those performed with ^177^Lu-DOTATOC in order to investigate the possible influence of conversion and Auger electrons of ^161^Tb on peptide disintegration.

**Results:**

Irradiations of enriched ^160^Gd targets yielded 6–20 GBq ^161^Tb. The final product was obtained at an activity concentration of 11–21 MBq/μL with ≥99% radionuclidic and radiochemical purity. The DOTA chelator was radiolabeled with ^161^Tb or ^177^Lu at the molar activity deemed useful for clinical application, even at the two-week time point after end of chemical separation. DOTATOC, radiolabeled with either ^161^Tb or ^177^Lu, was stable over 24 h in the presence of a stabilizer.

**Conclusions:**

In this study, it was shown that ^161^Tb can be produced in high activities using different irradiation facilities. The developed method for ^161^Tb separation from the target material yielded ^161^TbCl_3_ in quality suitable for high-specific radiolabeling, relevant for future clinical application.

**Electronic supplementary material:**

The online version of this article (10.1186/s41181-019-0063-6) contains supplementary material, which is available to authorized users.

## Background

The use of the β^−^–emitter ^177^Lu (E_β-av_ = 134 keV (100%), T_1/2_ = 6.7 d) (Solá 2000), in combination with somatostatin analogues (e.g. DOTATOC, DOTATATE), is considered a promising tool for the treatment of neuroendocrine tumors (NET). It has been extensively utilized in clinics, which recently resulted in the approval of Lutathera® (^177^Lu-DOTATATE), by the U.S. Food and Drug Administration (FDA) (Kam 2012; CHMP 2016). Treatment with ^177^Lu-DOTATATE resulted in longer progression-free survival time (65.2% at Month 20, compared to 10.8% in the control group); nevertheless, partial remission remained at ≤50% in the assessable patients, with the complete response being ≤12% (CHMP 2016; Strosberg 2017; Kwekkeboom 2005; van Essen 2007; Sansovini 2013). The radiolanthanide ^161^Tb shows similar decay characteristics (E_β-av_ = 154 keV (100%), T_1/2_ = 6.9 d (Solá 2000)) and coordination chemistry to ^177^Lu. ^161^Tb can, therefore, be stably coordinated with a DOTA chelator and be used in combination with a number of small molecules, peptides and antibodies currently employed with ^177^Lu. ^161^Tb may show an increased therapeutic efficacy over ^177^Lu, due to the co-emission of a substantially larger number of conversion and Auger electrons at a favorable energy range (~12 e^−^, ~36 keV per decay for ^161^Tb and ~ 1 e^−^, ~ 1.0 keV per decay for ^177^Lu, respectively) (Eckerman 2008; Müller 2014). The possibility of using ^161^Tb as an alternative to ^177^Lu was first proposed by Lehenberger et al. (Lehenberger 2011) and, subsequently, corroborated by Müller et al. by comparison of in vitro and in vivo studies using a DOTA-folate conjugate labeled with ^161^Tb and ^177^Lu (Müller 2014). The enhanced anti-tumor effect, as well as higher average survival time, was found in mice treated with ^161^Tb-folate over those which received ^177^Lu-folate. In a preliminary therapy study using ^161^Tb-PSMA-617, PSMA-positive PC-3 PIP tumor-bearing mice demonstrated significant tumor-growth delay, as compared to the control group, without causing early side effects (Müller 2018a). Better therapeutic efficacy was also observed for a ^161^Tb-labeled radioimmunoconjugate in an ovarian cancer model when compared to the ^177^Lu-radioimmunoconjugate counterpart (Grünberg 2014). The low-energy conversion and Auger electron emission from ^161^Tb contribute 26% – 88% to the total absorbed dose (compared to 10% – 34% for ^177^Lu), depending on the tumor size, which could be associated to its enhanced therapeutic efficacy over that of ^177^Lu (Champion 2016). The doses delivered by ^161^Tb or ^177^Lu to 10 mm-diameter spheres were calculated to be comparable for both radionuclides, however, for 100 μm-diameter and 10 μm-diameter spheres ^161^Tb could deliver 1.8 and 3.6 times higher dose than ^177^Lu, respectively, making ^161^Tb the more appropriate candidate for treating micrometastases (Hindie 2016). Also, the co-emission of 48.9 keV and 74.6 keV ^161^Tb γ-rays allows for the acquisition of single photon emission computed tomography (SPECT) images for dosimetry determination before administration of the therapeutic dose, comparable to that performed with ^177^Lu (Marin 2018). In addition, ^161^Tb could be used in combination with diagnostic radioisotopes, namely, ^152^Tb (PET) or ^155^Tb (SPECT) as a matched pair towards the concept of theragnostics (Müller 2012; Müller 2018b).

The ^161^Tb production route was proposed by Lehenberger et al. via the ^160^Gd(n,γ)^161^Gd → ^161^Tb nuclear reaction, which provided no-carrier-added radiolanthanide at high specific activities (~ 4 TBq/mg) (Lehenberger 2011). Enriched ^160^Gd(NO_3_)_3_ targets (ampoules) were prepared by dissolving ^160^Gd_2_O_3_ in nitric acid and evaporating to dryness. Lanthanide nitrates are hygroscopic materials, however, and the heating of the ampoule in the nuclear reactor (due to γ-rays from the reactor and β -rays generated in the sample) can create water vapor within the ampoule. The vapor could create overpressure, resulting in ampoule breakage. The ^161^Tb separation method from ^160^Gd(NO_3_)_3_ targets was previously developed at Paul Scherrer Institute (Villigen-PSI, Switzerland), but the radiolabeling capability of the ^161^Tb product was three times lower than the commercial no-carrier-added ^177^Lu (Lehenberger 2011). This implied that the ^161^Tb product contained undesired environmental impurities at the end of separation (EOS), thereby, compromising the capability of reproducible routine production.

Herein, we report on the large-scale ^161^Tb production from ^160^Gd_2_O_3_ target material, suitable for introduction into a process in accordance with Good Manufacturing Practice (GMP) and, thereafter, clinical application. The ^161^Tb purification method was improved by optimization of the Tb/Gd separation process, followed by characterization of the final product (^161^TbCl_3_). The ^161^Tb purity was compared with no-carrier-added ^177^Lu (EndolucinBeta), as currently produced by ITG GmbH, Germany, for worldwide clinical application.

## Methods

### Target preparation for the production of ^161^Tb

Gadolinium oxide (^160^Gd_2_O_3_, 98.2% enrichment, Isoflex, USA) was used as target material for the production of no-carrier-added ^161^Tb, as previously reported (Lehenberger 2011). The elemental composition of the target material in question is provided in the Supplementary Material (Table S1). To prepare the targets for irradiation at the spallation-induced neutron source (SINQ, Paul Scherrer Institute, 4.10^13^ n.cm^− 2^.s^− 1^), 80–95 mg ^160^Gd(NO_3_)_3_ were placed in a quartz glass ampoule (Suprasil, Heraeus, Germany) and sealed. Ampoules containing 7–33 mg ^160^Gd_2_O_3_ were prepared in a similar manner and sent for irradiation to two research nuclear reactors (SAFARI-1, South African Nuclear Energy Corporation, 2.10^14^ n.cm^− 2^.s^− 1^; and RHF ILL, Institut Laue–Langevin, 1.10^15^ n.cm^− 2^.s^− 1^). The mass of the target material, required for the irradiation at the chosen facility, was calculated using the ChainSolver code (Romanov 2005).

### Determination of the neutron fluxes of the irradiation facilities with ^59^Co monitors

In order to monitor neutron fluxes at ILL, SAFARI-1 and SINQ, quartz ampoules containing ^59^Co as standard (^59^Co in 2% w/w HNO_3_, Sigma-Aldrich, USA) with 33 ng – 2 μg ^59^Co (mass determined based on the volume of the standard solution pipetted) were prepared. Ampoules were dried at 80 °C, to ensure water evaporation, and sealed. Ampoules with ^59^Co standard were placed and sealed in the same Al container as the ampoules containing ^160^Gd target material, along with empty ampoules (used as references) for the irradiation process. ^59^Co masses were calculated to produce 50–100 kBq ^60^Co activity via ^59^Co(n,γ)^60^Co nuclear reaction, depending on the reactor neutron flux. The ^60^Co activities in the ampoules were measured after irradiations using a high-purity germanium (HPGe) detector (Canberra, France), in combination with the InterWinner software package (version 7.1, Itech Instruments, France), with a statistical uncertainty less than 5%. After irradiation at the facility in question and gamma spectrometry of the ampoules, the activities of ^60^Co produced by the added ^59^Co standards were determined by subtraction of the ^60^Co activities of the reference (empty) ampoules which stem from traces of cobalt impurities in the used quartz. Based on these ^60^Co activity values, the average neutron flux (*ϕ*_*th*_) of each irradiation was calculated (Table 1) using the following equation:

1 $$ {\phi}_{th}=\frac{A_0}{\sigma_n\bullet N\bullet \left(1-{e}^{-\lambda \bullet {t}_B}\right)} $$

where *A*_0_ is the ^60^Co activity at the end of bombardment, *σ*_*n*_ the thermal neutron capture cross section (37.18 ± 0.06 b (Mughabghab 2018)), *N* the number of target atoms, *λ* the radioactive decay constant and *t*_*B*_ the irradiation time.

**Table 1:** Measured neutron fluxes of the irradiation facilities used for ^161^Tb production

Facility	Irradiation time, d	^60^Co activity, kBq	Measured perturbed neutron flux, n.cm^− 2^.s^− 1^	Nominal unperturbed neutron flux, n.cm^− 2^.s^− 1^
ILL	10	33.5	7.4·10^14^	1.0·10^15^
SAFARI-1	13–16	41.0–54.0	1.8·10^14^	2.0·10^14^
SINQ	21	92.3	1.8·10^13 a^	4.0·10^13^

^a^Measurement performed while spallation target was operated at lower than optimum beam current

### Development of the procedure for the ^161^Tb purification process

A chromatographic column (10 mm × 170 mm) was prepared using Sykam macroporous cation exchange resin (Sykam Chromatographie Vertriebs GmbH, Germany; particle size 12–22 μm, NH_4_^+^ form). The separation parameters were first optimized by means of bench experiments with the use of radioactive tracers (Additional file 1: Table S2) and subsequently applied towards the separation of an aliquot of reactor-produced ^161^Tb (230 MBq). These experiments resulted in the design and construction of a chemical separation module, such that high activities (GBq) of the radionuclide can be processed in the hot cell. The quartz glass ampoule with the ^160^Gd_2_O_3_ target material, delivered from the irradiation facility, was placed in a plastic target tube, crushed and attached to the module inside the hot cell with the aid of manipulators. The target material from the ampoule was dissolved in 2.0 mL 7.0 M nitric acid (HNO_3_, Suprapur, Merck, Germany), followed by evaporation at 80 °C under nitrogen flow. The residue was taken up in 0.1 M ammonium nitrate (prepared from 25% Suprapur NH_3_ and 65% Suprapur HNO_3_, Merck, Germany) and loaded onto the cation exchange resin column. The ^161^Tb separation from the target material and impurities was performed with the use of 0.13 M (pH 4.5) α-hydroxy-isobutyric acid (α-HIBA, Sigma-Aldrich GmbH, Germany) as eluent. Concentration of ^161^Tb was performed using the bis(2,4,4-trimethyl-1-pentyl) phosphinic acid extraction resin (LN3, Triskem International, France; 6 mm × 5 mm), followed by the elution of the final product (^161^TbCl_3_) in 500 μL 0.05 M hydrochloric acid (HCl, Suprapur, Merck, Germany). The pH of the final product was determined using pH indicator strips (Merck, Germany).

### Characterization of the ^161^Tb product after purification

#### Radionuclidic purity

The identification and radionuclidic purity of the ^161^Tb were examined by γ-ray spectrometry using the HPGe detector mentioned above. The aliquot of the final product, containing 5–10 MBq of ^161^Tb, was measured with the HPGe detector until the 3σ uncertainty was below 5%.

#### Radiochemical purity

The radiochemical purity of the final product was determined by means of radio thin layer chromatography (radio TLC) using a procedure established for ^177^Lu (Oliveira 2011). The aliquot of ^161^TbCl_3_ (2 μL, ~ 100 kBq) was deposited on TLC silica gel 60 F_254_ plates (Merck, Italy) and placed in the chamber with 0.1 M sodium citrate solution (pH 5.5; Merck, Germany) mobile phase. After elution, the plate was dried and analyzed using a radio TLC scanner instrument (Raytest Isotopenmessgeräte GmbH, Germany). The results were interpreted with the MiniGita Control software package (version 1.14, Raytest Isotopenmessgeräte GmbH, Germany).

#### Radiolabeling yield

Radiolabeling of DOTANOC (ABX GmbH, Germany) at a molar activity of 180 MBq/nmol (1-to-4 nuclide-to-peptide molar ratio) was performed in order to evaluate the success of the purification process. Sodium acetate (Alfa Aesar, Germany; 0.5 M, pH 8) was added to ^161^TbCl_3_ solution (~ 200 MBq) to adjust pH to ~ 4.5. The relevant quantity of DOTANOC was subsequently added from a 1 mM stock solution. The reaction solution was incubated for 10 min at 95 °C. The radiolabeling yield was determined by reverse-phase high performance liquid chromatography (HPLC, Merck Hitachi LaChrom) with a radioactivity detector (LB 506, Berthold, Germany) and a C-18 reverse-phase column (150 mm × 4.6 mm; Xterra™ MS, C18; Waters). Trifluoroacetic acid 0.1% (Sigma-Aldrich, USA) in MilliQ water (A) and acetonitrile (VWR Chemicals, USA; HPLC grade) (B) were used as mobile phase with a linear gradient of solvent A (95–5% over 15 min) in solvent B at a flow rate of 1 mL/min. The sample for the analysis was prepared by diluting ~ 0.3 MBq aliquot of the radiolabeling solution in 100 μL MilliQ water containing sodium diethylenetriamine pentaacetic acid (Na-DTPA, 50 μM). The radiolabeling yield of ^161^Tb-DOTANOC was determined by integration of the product peak from the obtained HPLC chromatogram in relation to the sum of all radioactive peaks (the radiolabeled product, potentially released ^161^Tb as well as degradation products of unknown structure), which were set to 100%.

### Determination of the radiolabeling yield of ^161^Tb- and ^177^Lu-DOTA over a two-week period

In order to assess the change of the molar activity of ^161^Tb-DOTA at different DOTA-to-nuclide molar ratios over time (2 weeks after EOS) and to compare it with the molar activity of ^177^Lu-DOTA, thin layer chromatography (TLC) analysis was performed. The required DOTA solutions (1–500 pmol DOTA) were obtained by dilution of the initial 1 mM DOTA solution (CheMatech, France) with 0.5 M sodium acetate (pH 4.5). The prepared DOTA dilutions were mixed with 2.5–4 MBq ^161^Tb (corresponding to 3.1–5 pmol), ^177^Lu (no-carrier-added, ITG GmbH, Germany) or ^177^Lu (carrier-added, IDB Holland bv, the Netherlands) at different DOTA-to-nuclide molar ratios (160:1 to 1:1). The reaction solutions were incubated for 20 min at 95 °C and 2 μL of each solution were deposited on TLC silica gel 60 F_254_ plates, which served as a stationary phase. The mixture of 10% ammonium acetate (Sigma-Aldrich, USA) and methanol (Merck, Germany) was used as mobile phase (ratio 1:1 (*v/v*), pH 5.5). After elution, the phosphor screen (Multisensitive, Perkin Elmer Inc., USA) was illuminated with the TLC plate and analyzed with a Cyclon Phosphor Imager (Perkin Elmer Inc., USA). The peaks corresponding to the unlabeled ^161^Tb or ^177^Lu (R_f_ = 0) and to the ^161^Tb- or ^177^Lu-DOTA compounds (R_f_ = 0.4) were integrated with the OptiQuant image analysis software (version 5.0, Perkin Elmer Inc., USA) and the radiolabeling yield determined. Based on the data obtained, the DOTA-to-nuclide molar ratios were plotted against the radiolabeling yield using Origin software, fitted with a Boltzmann’s sigmoidal modified equation. Experiments were repeated three times for ^161^Tb and ^177^Lu (no-carrier-added) and once for ^177^Lu (carrier-added). The average DOTA-to-nuclide molar ratios, corresponding to 50% labeling efficiency of DOTA with ^161^Tb (no-carrier-added) and ^177^Lu (no-carrier-added or carrier-added) at different time points (Day 3 to Day 14 after EOS), were determined and compared with each other for statistical significance by an unpaired t test using Graph Pad Prism (version 7.00).

### ^161^Tb/^177^Lu-DOTATOC stability studies

The radiolabeling of DOTATOC with no-carrier-added ^161^Tb or no-carrier-added ^177^Lu at 50 MBq/nmol molar activity (300 MBq ^161^Tb activity in total) was performed as described above, in the absence or in the presence of L-ascorbic acid (2.9 mg, Sigma-Aldrich, USA). The radiolabeling yield was determined by means of HPLC (as described above) immediately after the preparation of ^161^Tb/^177^Lu-DOTATOC. The radioactivity concentration of the labeling solutions was adjusted to 250 MBq/500 μL with saline and radiolytic stability of the radioligand was determined over time (1 h, 4 h and 24 h) by means of HPLC.

## Results

### ^161^Tb production yield (theoretical versus experimental)

No-carrier-added ^161^Tb was produced by neutron irradiation of enriched ^160^Gd_2_O_3_ (98.2% enrichment) targets via the ^160^Gd(n,γ)^161^Gd → ^161^Tb nuclear reaction. The mass of the target material had to be calculated precisely in order to ensure that the ^161^Tb activity allowed for international transportation was not exceeded. ^161^Tb is not explicitly listed in the dangerous goods tables of the ADR (European Agreement concerning the International Carriage of Dangerous Goods by Road) and International Air Transport Agency (IATA) regulations which are, in turn, based on IAEA (International Atomic Energy Agency) recommendations. As a result, the generic A2 value (activity limit of radioactive material) according to the “Basic Radionuclide Values for Unknown Radionuclides or Mixtures” of 0.02 TBq has to be applied (IAEA 2012). Due to this restriction, a maximum of 20 GBq ^161^Tb could be transported internationally (in this case, shipping from ILL and SAFARI-1 research reactors to PSI) (IAEA 2012). Masses of the ^160^Gd target material, required to produce 20 GBq ^161^Tb after bombardment at the irradiation facilities, were calculated with the ChainSolver code using the recommended cross-section for thermal neutron capture on ^160^Gd of 1.4(3) b (Mughabghab 2018). Two-week irradiations at ILL (6.5 mg ^160^Gd_2_O_3_, 1.10^15^ n.cm^− 2^.s^− 1^, 1 day cooling) and at SAFARI-1 (32 mg ^160^Gd_2_O_3_, 2.10^14^ n.cm^− 2^.s^− 1^, 1 day cooling) would theoretically result in 20 GBq ^161^Tb. At PSI’s neutron source facility (SINQ, 4·10^13^ n.cm^− 2^.s^− 1^) each irradiation cycle is 3 weeks, which was calculated to provide 17.2 GBq ^161^Tb after the bombardment of 100 mg ^160^Gd(NO_3_)_3_. The masses of the target material could be adapted to operator/user requirements based on the ChainSolver code calculations and neutron fluxes, calculated from the measured ^60^Co activity values of the ^59^Co monitors (Table 1). Three ampoules with ^59^Co were bombarded at the SAFARI-1 nuclear reactor and one ampoule each at ILL and at SINQ irradiation facilities (together with the ^160^Gd ampoules), respectively. The measured values of the perturbed neutron fluxes in the samples irradiated at the ILL and SAFARI-1 nuclear reactors scaled as expected with the unperturbed neutron flux values reported by the facility in question.

In practice, one-to-two-week irradiations of ^160^Gd target ampoules using SAFARI-1 (22–33 mg ^160^Gd_2_O_3_) and ILL (7–13 mg ^160^Gd_2_O_3_) research reactors resulted in production of 10–20 GBq of ^161^Tb (Table 2). The calculated neutron flux at the PNA irradiation position of the SINQ facility was determined experimentally to be only ~ 50% of its originally reported value of 4 10^13^ n.cm^− 2^.s^− 1^ (Table 1), which resulted in 6–9 GBq ^161^Tb after 3 weeks irradiation of the enriched ^160^Gd(NO_3_)_3_ target material (Table 2). This was due to the fact that the spallation target had recently been replaced and was being operated at a lower beam current (1.3 mA protons instead of 2.3 mA) than normally specified (Table 1).

**Table 2:** Production yields of several ^161^Tb batches, obtained from the irradiation facilities

Facility	Irradiation time, d	Target material	Mass of the target material, mg	Measured ^161^Tb activity (EOB), GBq
ILL	5	^160^Gd_2_O_3_	12.5	11.6
ILL	10	^160^Gd_2_O_3_	7.3	16.7
SAFARI-1	14	^160^Gd_2_O_3_	33.3	19.6
SAFARI-1	7	^160^Gd_2_O_3_	32.5	11.9
SINQ	21	^160^Gd(NO_3_)_3_	94.9	8.8
SINQ	21	^160^Gd(NO_3_)_3_	86.4	6.0

### Radiochemical separation of ^161^Tb from the target material and accumulated impurities

**Fig. 1:**
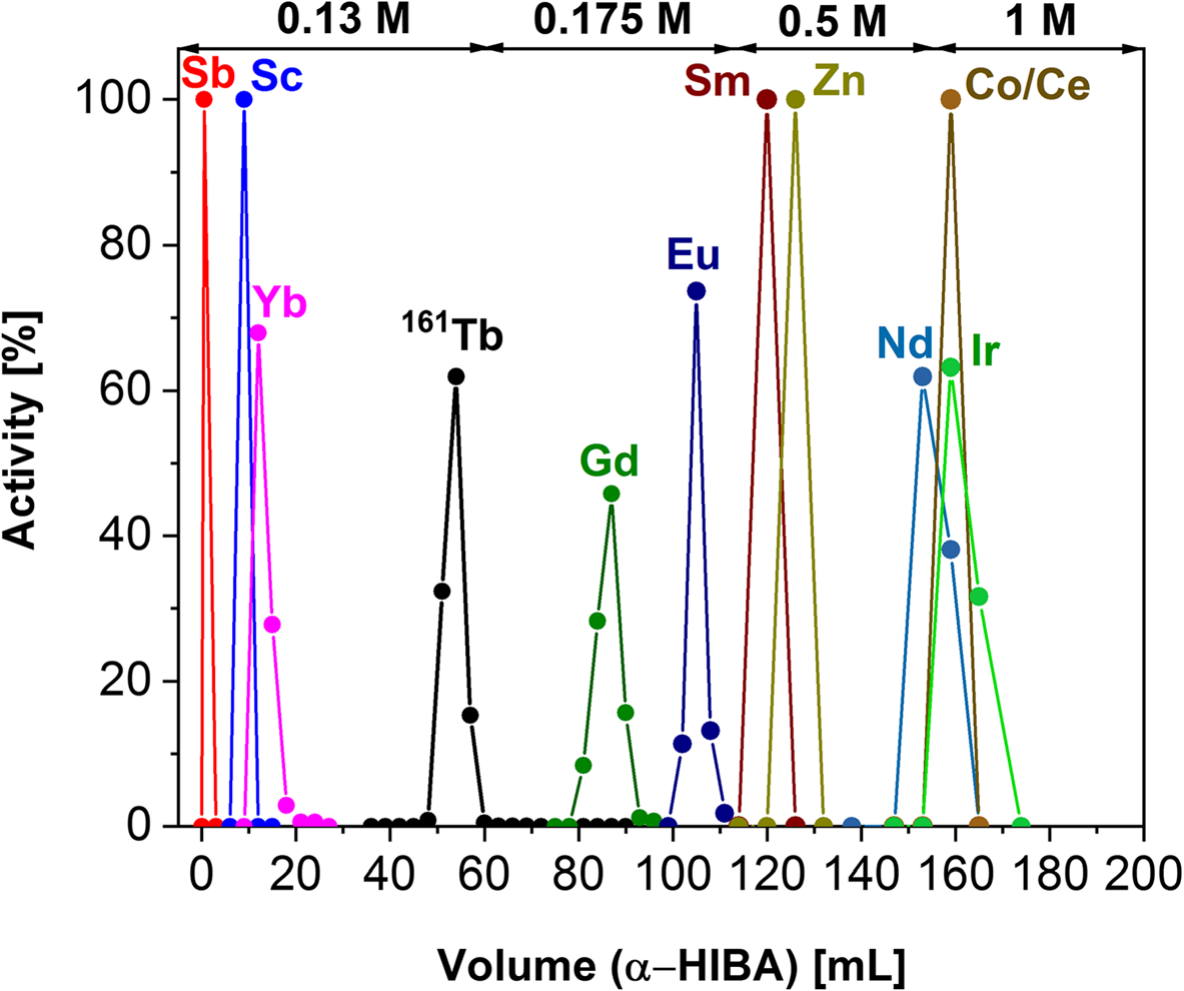
Elution profile of ^161^Tb separation from the irradiated target material and side products (10 mm × 170 mm Sykam resin column, 8 mg ^160^Gd_2_O_3_, 0.6 mL/min eluent flow rate)

By performing bench experiments using Sykam cation exchange resin column (10 mm × 170 mm) and long-lived radioactive tracers, conditions for the efficient separation of Tb from up to 140 mg of Gd_2_O_3_ and the presence of various impurities were established (Additional file 1: Figure S1 and S2). Subsequently, the established experimental conditions (Sykam resin; 0.13 M α-HIBA, pH 4.5; 0.6 mL/min eluent flow rate) were applied towards the purification of the reactor-produced ^161^Tb (230 MBq). During the irradiation, radioactive side products were co-produced from the impurities of the target material (^46^Sc, ^124^Sb, ^141^Ce, ^147^Nd, ^153^Gd, ^153^Sm, ^152/154/155/156^Eu, ^169^Yb) and the ampoule material (^65^Zn, ^60^Co, ^192^Ir). Despite this impurity formation, the established method demonstrated effective ^161^Tb separation from Gd target material and the impurities using the Sykam resin column (Fig. 1). ^161^Tb was eluted from the Sykam resin with 20 mL α-HIBA, followed by the concentration of the radionuclide on the LN3 resin column (Additional file 1: Figure S3). LN3 extraction resin was reported to have low affinity for Tb ions in low concentrated acids (McAlister 2007), thereby, allowing ^161^Tb to be eluted in a small volume of 0.05 M HCl.

**Fig. 2:**
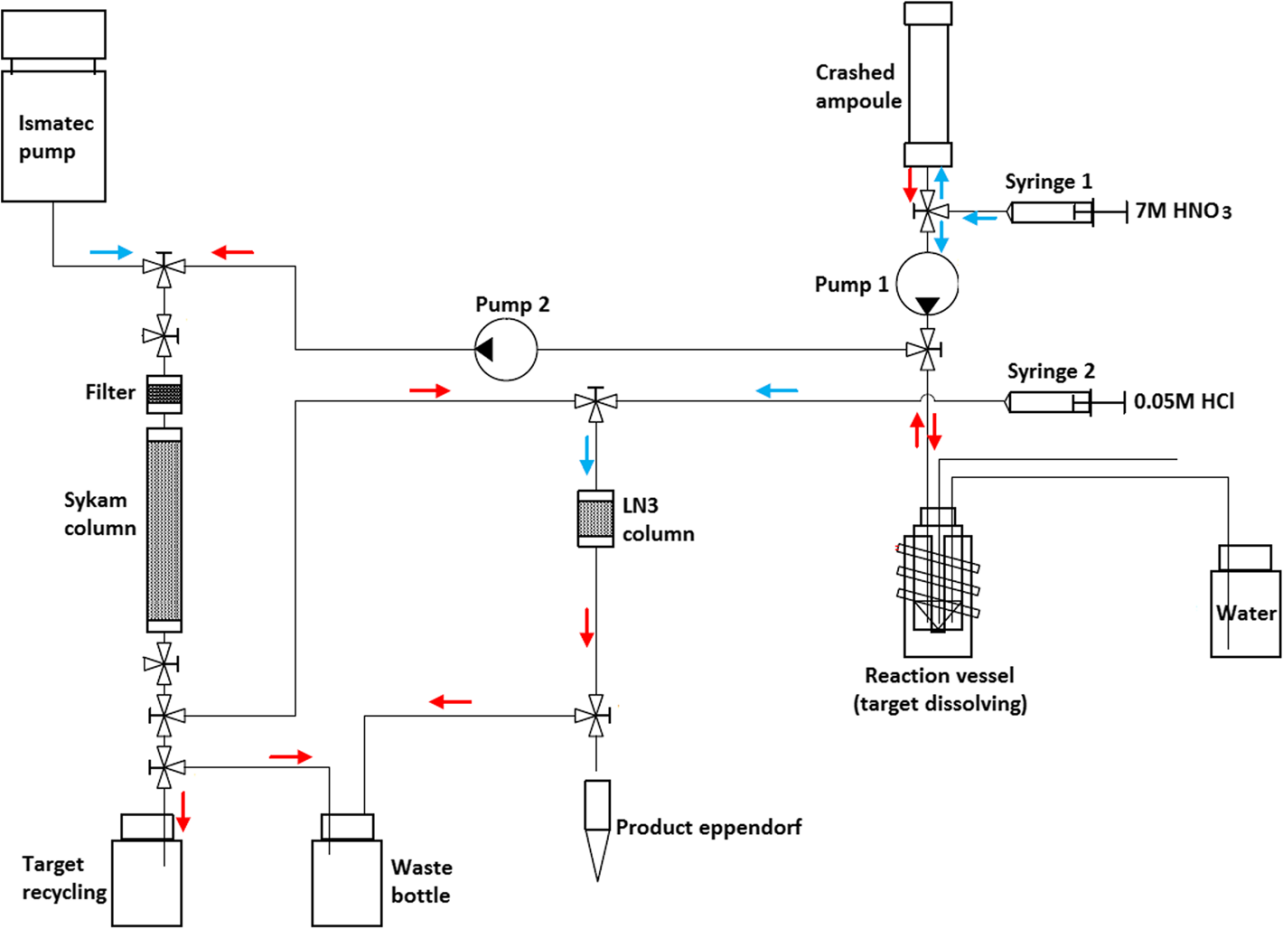
Schematic diagram of the ^161^Tb chemical separation system

Based on the developed two-column purification method (combination of Sykam and LN3 resin columns), a ^161^Tb purification module was designed (Fig. 2). The module was constructed and placed inside the hot cell, making it possible to perform separations with higher activities (up to 20 GBq) of the reactor-produced ^161^Tb.

The established procedure for the ^161^Tb purification process using the designed module resulted in the elution of the final product (^161^TbCl_3_) in a small volume (500 μL) of 0.05 M HCl, with an activity concentration of 11–21 MBq/μL. A separation yield of 80–90% was achieved at EOS. Losses of 10–20% of ^161^Tb activity were observed in the target dissolving, column loading and final elution steps.

### Characteristics of the ^161^TbCl_3_ product

**Fig. 3:**
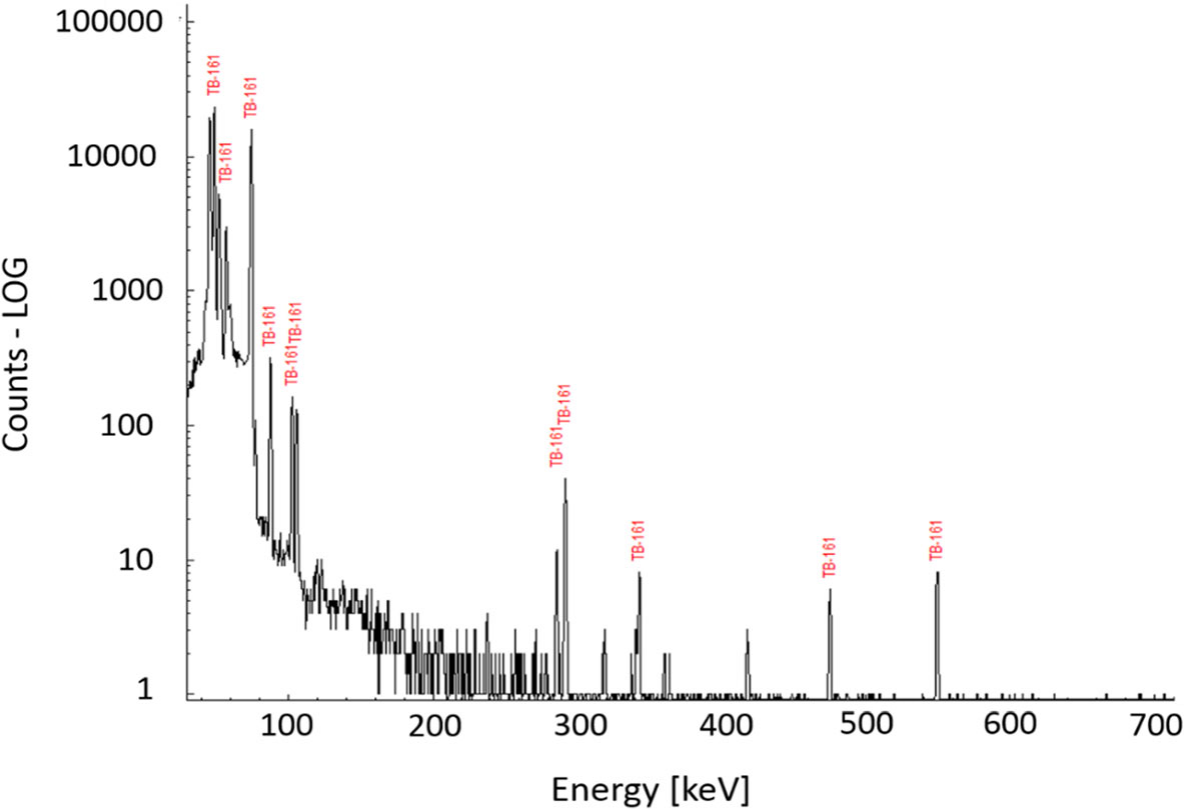
Gamma spectrum of ^161^Tb, obtained after the purification process. The radionuclidic impurity ^160^Tb is not visible due to the negligible activity as compared to that of ^161^Tb at end of separation (EOS)

**Fig. 4:**
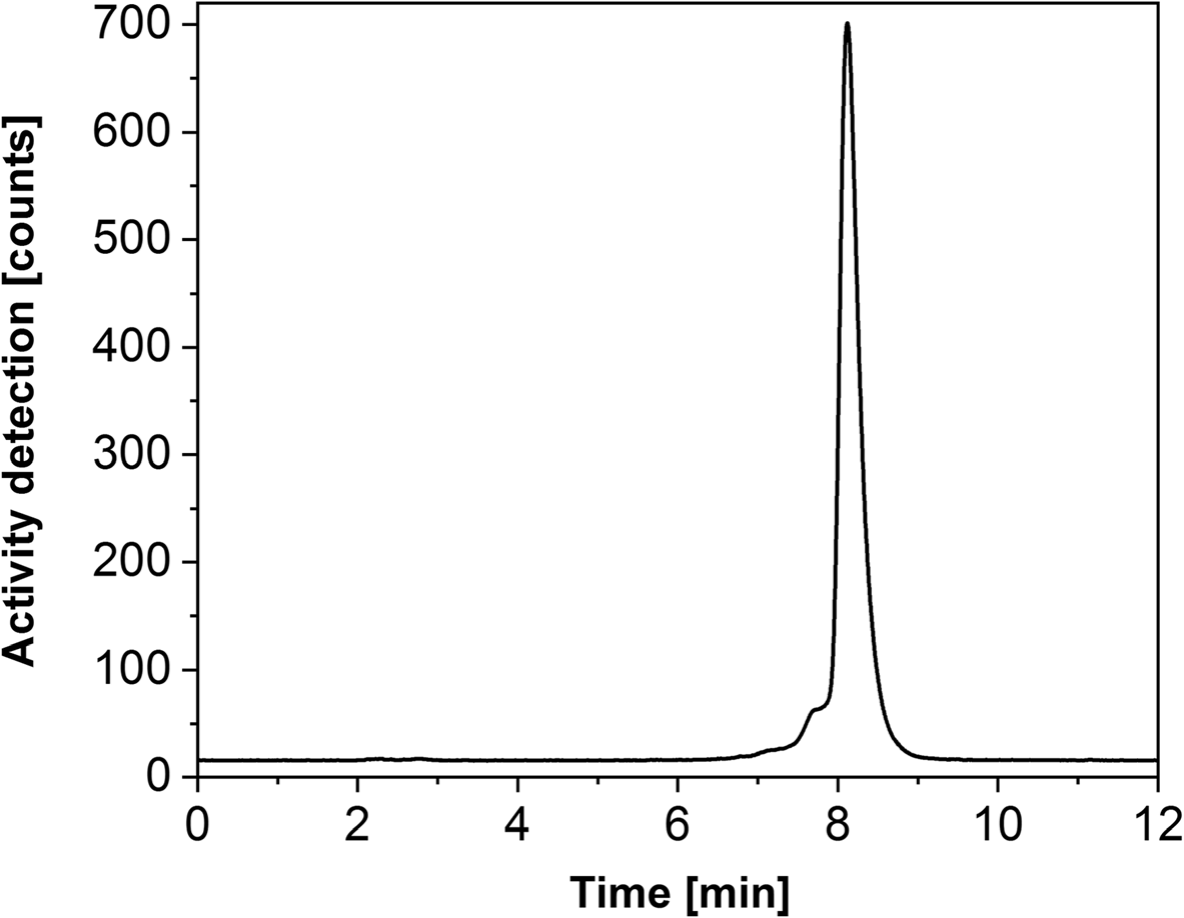
HPLC chromatogram of ^161^Tb-DOTANOC (2.3 min retention time would indicate “free” or unlabeled ^161^Tb, while 8.2 min indicates ^161^Tb-DOTANOC)

^161^Tb, obtained after the purification process, was characterized to provide a product specification (Table 3). The identification of the product was confirmed by the ^161^Tb-characteristic γ-lines (Fig. 3). The content of long-lived ^160^Tb (T_1/2_ = 72.3 d), produced by the ^159^Tb(n,γ)^160^Tb nuclear reaction due to the presence of ^159^Tb impurity in the target material (as sold by the vendor), was determined after the decay of ^161^Tb and did not exceed 0.007% of the total ^161^Tb activity at EOS (Additional file 1: Figure S4). The radiochemical purity of the ^161^TbCl_3_, determined using radio-TLC, was > 99% (Additional file 1: Figure S5). The radiolabeling yield of ^161^Tb-DOTANOC showed ≥99% efficiency at 180 MBq/nmol molar activity, which corresponds to 1-to-4 nuclide-to-peptide molar ratio (Fig. 4).

**Table 3:** Product data specification of ^161^TbCl_3_, developed in this work, compared to the commercially-available ^177^LuCl_3_

Test	Specification of ^161^TbCl_3_	Specification of ^177^LuCl_3_ (EndolucinBeta)^a^
Radioactivity concentration	11–21 MBq/μL	36–44 MBq/μL
Appearance	Clear and colorless solution	Clear and colorless solution
pH	1–2	1–2
Radiolabeling yield HPLC based on radiolabeling with^161^Tb of DOTANOC, molar ratio 1:4 (180 MBq/nmol)	> 99%	> 99%
Identity ^161^Tb (γ-ray spectrometry)	48.9 keV γ-line 74.6 keV γ-line	113 keV γ-line 208 keV γ-line
Radionuclidic purity (γ-ray spectrometry)	^160^Tb ≤ 0.007%	^175^Yb ≤ 0.01%
Radiochemical purity (radio-TLC)	> 99%	> 99%

^a^Specification from the EndolucinBeta certificate of analysis (ITG)

### Comparison of the ^161^Tb and ^177^Lu quality, based on the ^161^Tb- and ^177^Lu-DOTA molar activities over a two-week period

**Fig. 5:**
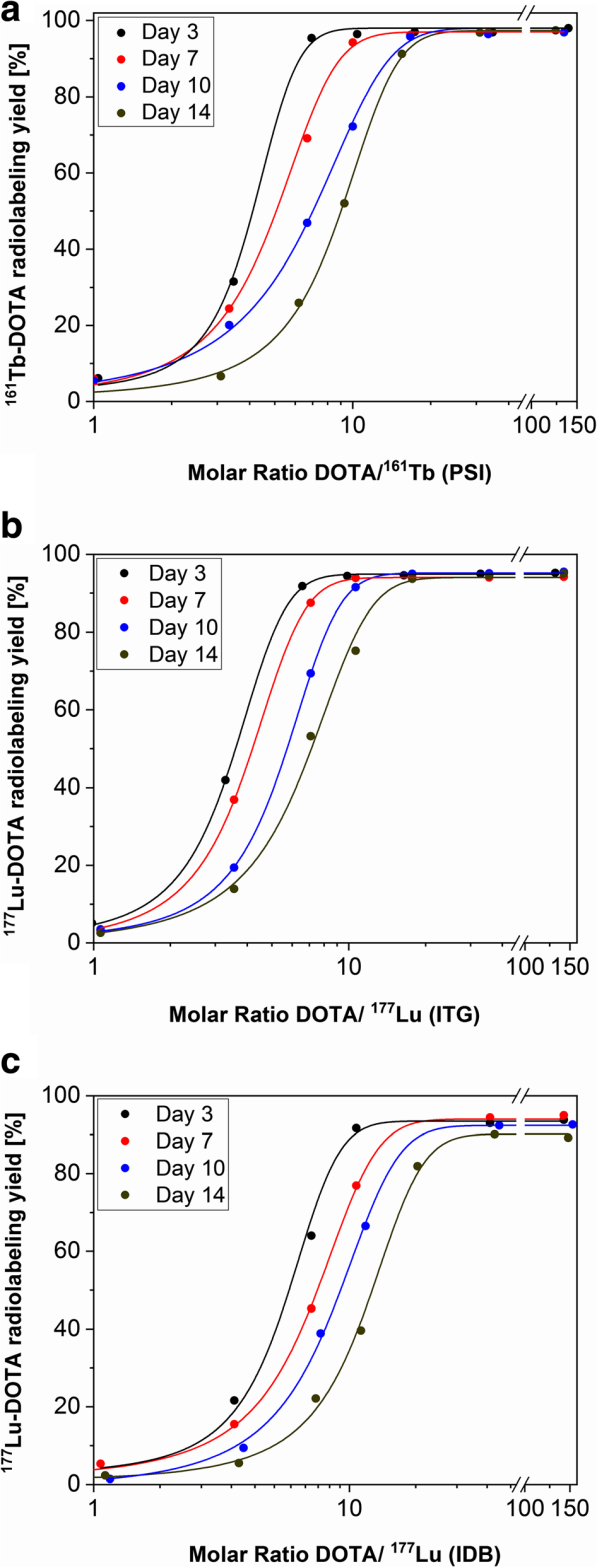
Comparison of the radiolabeling yield of (**a**) no-carrier-added ^161^Tb (SAFARI-1); **b** no-carrier-added ^177^Lu (ITG) and (**c**) carrier-added ^177^Lu (IDB) in combination with DOTA over time at different DOTA-to-nuclide molar ratios

Radiolabeling of DOTA with ^161^Tb (no-carrier-added) and ^177^Lu (either carrier-added or no-carrier-added) was performed at different DOTA-to-nuclide molar ratios in order to monitor the quality change of the radiolanthanides of interest over a two-week period after EOS. DOTA could be complexed with ^161^Tb and ^177^Lu (no-carrier-added) at 15:1 and 13:1 DOTA-to-nuclide molar ratios, respectively, with > 90% radiolabeling yield at Day 14 after EOS (Fig. 5 a, b). This indicates the possibility of using the prospective drug product (e.g. DOTA peptides radiolabeled with ^161^Tb) for up to 2 weeks after the chemical separation. With carrier-added ^177^Lu, 90% radiolabeling yield was only achieved when using a much higher DOTA-to-nuclide radio (32:1) at the two-week time point (Fig. 5c).

The average DOTA-to-nuclide molar ratios corresponding to 50% labeling efficiency of DOTA with ^161^Tb and ^177^Lu were determined at specific time points (Day 3, Day 7, Day 10 and Day 14 – Table 4) (Additional file 1: Figure S7). The values allow an estimation of the possible radiolabeling yield of different biomolecules conjugated with a DOTA chelator, labeled with the radionuclide of interest, over a certain decay period, as well as comparison of the radiolabeling capability of the radionuclides of interest. When DOTA was radiolabeled with carrier-added ^177^Lu, 50% labeling efficiency was obtained at higher DOTA-to-nuclide molar ratios at each time point as compared to no-carrier-added ^161^Tb and ^177^Lu, respectively. The 50% labeling efficiency of ^161^Tb-DOTA was found to be comparable with that of no-carrier-added ^177^Lu at Day 3 (*p* = 0.13), while a slight increase of the values was observed for Day 7, Day 10 and Day 14 (*p* > 0.05), respectively.

**Table 4:** DOTA-to-nuclide molar ratios, corresponding to 50% labeling efficiency of ^161^Tb-DOTA and both carrier-added and no-carrier-added ^177^Lu-DOTA over a two-week decay period

Nuclide	Day 3	Day 7	Day 10	Day 14
^161^Tb (PSI) *No-carrier-added*	4.4 ± 0.3	5.7 ± 0.4	7.6 ± 0.5	9.4 ± 0.3
^177^Lu (ITG) *No-carrier-added*	3.8 ± 0.1	4.2 ± 0.1	5.7 ± 0.1	7.1 ± 0.1
^177^Lu (IDB) *Carrier-added*	5.8*	7.5*	9.2*	12.2*

* Statistically different to no-carrier-added ^161^Tb and ^177^Lu (*p* < 0.05)

### Radiolytic stability of ^161^Tb-DOTATOC in comparison to ^177^Lu-DOTATOC

**Fig. 6:**
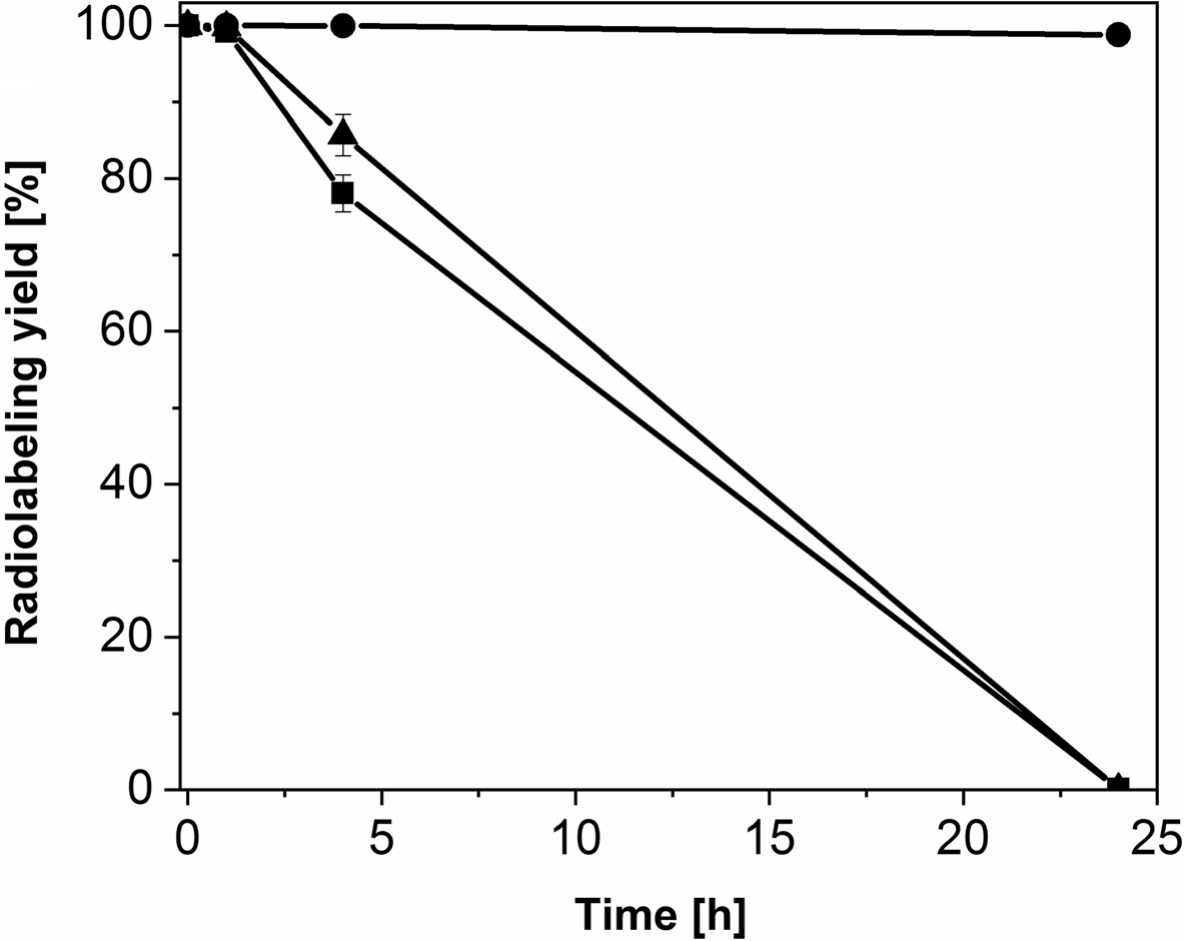
Stability of the radiopeptide over time, in the presence and absence of ascorbic acid: the graph shows the % intact ^161^Tb/^177^Lu-DOTATOC over a period of 24 h. In the presence of ascorbic acid ^177^Lu and ^161^Tb-DOTATOC curves overlap, making it impossible to visualize both simultaneously

In order to determine whether the conversion and Auger electrons from ^161^Tb may cause additional radiolytic degradation of radiolabeled somatostatin analogues, stability tests were performed using DOTATOC. The preparation of the any clinically-applied radiopharmaceutical (e.g. ^177^Lu-DOTATOC, ^177^Lu-DOTATATE), requires the use of a stabilizer (e.g. L-ascorbic acid, gentisic acid) in order to inhibit peptide autoradiolysis (LUTATHERA n.d.; Mukherjee et al. 2015; Liu et al. 2003; Dash 2015; Esser 2006; Schuchardt 2013). The stability studies of ^161^Tb-DOTATOC and ^177^Lu-DOTATOC were performed with and without addition of L-ascorbic acid. Both ^161^Tb- and ^177^Lu-DOTATOC were stable over 24 h incubation in the presence of a stabilizer (L-ascorbic acid) and showed > 98% radiolabeling yield at the 24-h time point. ^161^Tb-DOTATOC and ^177^Lu-DOTATOC were stable over 1 h (> 99% intact product) in the absence of L-ascorbic acid, but both showed radiolytic degradation after an incubation period of 24 h. After 4 h, slight degradation of both ^161^Tb-DOTATOC (78% intact product) and ^177^Lu-DOTATOC (85% intact product) was observed (Fig. 6). No significant influence of conversion and Auger electrons from ^161^Tb on radioligand stability was observed, when compared to ^177^Lu.

## Discussion

In the present study, the development of a reproducible chemical separation to produce no-carrier-added ^161^Tb from enriched ^160^Gd targets and the characterization of the final product (^161^TbCl_3_) is reported. Gd (NO_3_)_3_, previously used as target material (Lehenberger 2011), is not suitable for large-scale ^161^Tb production as lanthanide nitrates are hygroscopic materials, which begin to decompose at 70 °C (Fukuda 2018; Kalekar 2017). The pressure change due to the water evaporation and release of the gases at higher temperatures inside the ampoule may result in the ampoule cracking. The use of oxide targets (melting point 2420 °C) eliminates the potential issues described and will potentially give rise to large-scale ^161^Tb production in future (TBq activity). The ChainSolver code allows for quick calculations of the required masses of ^160^Gd targets for the production of the desired ^161^Tb activity, based on irradiation times and neutron fluxes, obtained with ^59^Co monitors.

Another big advantage of the newly-devised method over that of the previously-developed one is that the ^161^Tb product is eluted from LN3 in a small volume and can be used directly for labeling, while the previous method required the use of evaporation after elution from AG50W-X8, thereby, increasing the risk of introduction of environmental contamination and reducing the quality of the final product. The use of LN3 also ensures elution of elements in reverse order when compared to the Sykam/α-HIBA system, therefore, any impurities that may have eluted with ^161^Tb before introduction to the second column will be eluted differently from the LN3 resin column.

Recently, Brezovcsik et al. reported a Tb separation procedure from massive Gd targets (> 100 mg) using a 20 cm long analytical HPLC column (GE), indicating no mass influence of Gd on the separation process (Brezovcsik 2018). The efficiency of Tb separation from Gd was only 85%, however, showing significant overlapping of Tb and Gd peaks, which would result in the presence of “cold” Gd in the Tb final product and significantly decrease radiolabeling efficiency. The purification method described in this work provides an effective ^161^Tb separation from the Gd_2_O_3_ target material of masses up to 140 mg. This is a valuable result for possible future commercial application of the developed method for ^161^Tb separation from massive Gd_2_O_3_ targets (> 100 mg). For example, 2 weeks irradiation of 160 mg ^160^Gd_2_O_3_ target in ILL's nuclear reactor could theoretically result in the production of 0.5 TBq ^161^Tb (ChainSolver Code calculations (Romanov 2005)) which can be efficiently purified with the investigated method. ^160^Gd_2_O_3_ target material contains various trace elements (Additional file 1: Table S2), which may show similar chemical behavior to ^161^Tb on the resin in question and result in the elution of Tb and the impurities in the same fraction. It was demonstrated that, despite the impurities that could be produced via activation of trace elements in the target material or in the quartz ampoule, the established purification method effectively separated the ^161^Tb from potential impurities present in the system, based on the combination of Sykam and LN3 resin columns.

The two-column ^161^Tb purification process proposed by Lehenberger et al. (Lehenberger 2011) was adopted as the baseline for this study towards further development. The cation exchange resin of the first column, column dimension and pump flow rate were changed, while the concentration and pH of the eluent remained the same (0.13 M α-HIBA pH 4.5). The resin used for the second column was changed from AG 50 W-X8 (cation exchange resin) to LN3 (extraction resin). These modifications mentioned above played a vital role in obtaining the final product (^161^TbCl_3_) in purity comparable to that of the commercially-available no-carrier-added ^177^LuCl_3_ (EndolucinBeta). The radiolabeling capability of ^161^Tb in this work was similar to no-carrier-added ^177^Lu and three times higher than that of ^161^Tb obtained by Lehenberger et al. (Lehenberger 2011). Somatostatin analogues could be labeled with no-carrier-added ^161^Tb (this work) at 1-to-4 nuclide-to-peptide molar ratio (Fig. 4) immediately after the purification process with > 99% radiolabeling yield. Quantitative formation of ^161^Tb-DOTATATE was possible only at 1-to-12 nuclide-to-peptide molar ratio (Lehenberger 2011).

As expected, the radiolabeling capability of the produced, no-carrier-added ^161^Tb was higher than the radiolabeling capability of carrier-added ^177^Lu at each time point over the two-week decay period after the chemical separation process. The faster drop of ^161^Tb radiolabeling capability as compared to no-carrier-added ^177^Lu at Day 7, Day 10 and Day 14 after purification (Table 4) could be explained by the lower radioactivity concentration of the final product obtained (11–21 MBq/μL for ^161^TbCl_3_ vs 36–44 MBq/μL for ^177^LuCl_3_). This implies that the mass ratio between ^161^Tb and the impurities (Zn, Fe, Co, Pb etc.), which can be introduced during product analysis and post-processing, is lower compared to ^177^Lu. This results in the potentially stronger interference from environmental impurities during radiolabeling of DOTA with ^161^Tb than with ^177^Lu (Asti 2012). Nevertheless, complexation of ^161^Tb with DOTA was possible at Day 14 (after radiochemical separation) at 1-to-15 nuclide-to-peptide molar ratio (corresponding to 48 MBq/nmol molar activity), with > 90% radiolabeling yield (Fig. 5). This ratio would be appropriate when using DOTA-functionalized targeting agents, such as peptides for peptide receptor radionuclide therapy (Dash 2015). These excellent achievements indicate the possible clinical use of ^161^TbCl_3_ for a period of up to 2 weeks after EOS. Moreover, clinically-applied DOTATOC radiolabeled with ^161^Tb was stable over 24 h at high radioactivity concentration, indicating that conversion and Auger electrons had no negative influence on the stability, hence, storage and transportation of ^161^Tb-labeled somatostatin analogues would be feasible, as is the case for their ^177^Lu-labeled counterparts.

## Conclusions

A new method to separate ^161^Tb from its enriched ^160^Gd_2_O_3_ target material and co-produced impurities was developed with the use of cation exchange and extraction chromatography, respectively. The method resulted in radionuclidically and radiochemically pure product (^161^TbCl_3_), comparable to commercially available, no-carrier-added ^177^Lu. The quantity and quality of ^161^Tb is suitable for high-specific radiolabeling, potentially useful for the GMP production of radioligands towards future clinical application.

## Additional file

Additional file 1: **Table S1.** Chemical admixtures of ^160^Gd_2_O_3_, provided by the supplier (Isoflex, USA). **Table S2.** Long-lived radioactive tracers used for bench experiments. **Figure S1.** Elution profile of Tb/Gd separation from the target material (10 mm x 170 mm Sykam resin column, 0.6 mL/min eluent flow rate). Experiment 1 (**a**) was run without addition of ^nat^Gd_2_O_3_, while Experiment 2 (**b**) was performed with the addition of 140 mg ^nat^Gd_2_O_3_. **Figure S2.** Elution profile of Tb separation from the target material (Gd) and the impurities of the target material (10 mm x 170 mm Sykam resin column, 0.6 mL/min eluent flow rate). Experiment 3 (**a**) ^65^Zn and ^22^Na were added to the system as impurities. Experiment 4 (**b**) ^59^Fe, ^51^Cr and ^65^Ni were added to the system as impurities. **Figure S3.** Elution profile of Tb separation from Cr as a possible radioactive impurity in the final ^161^Tb product (6 mm x 5mm LN3 resin column, 0.6 mL/min eluent flow rate). **Figure S4.** Gamma spectrum of the decayed product (^161^TbCl_3_) used for the determination of ^160^Tb radionuclidic impurity in the total ^161^Tb fraction. No other radionuclides, other than ^160^Tb were found. **Figure S5.** Radio TLC chromatogram of ^161^TbCl_3_ solution in 0.1 M sodium citrate (pH 5.5) for the determination of ^161^Tb radiochemical purity. (DOCX 911 kb)

## Data Availability

The dataset(s) supporting the conclusions of this article is (are) included within the article (and its additional file(s)).

## Abbreviations

EOI: End of Irradiation
EOS: End of Separation
GMP: Good Manufacturing Practice
HPGe: High-purity Germanium
HPLC: High Performance Liquid Chromatography
IATA: International Air Transport Agency
PET: Positron Emission Tomography
SPECT: Single Photon Emission Computed Tomography
TLC: Thin Layer Chromatography
α-HIBA: α-hydroxy-isobutyric acid
