# Supplementary material for: Production and characterization of no-carrier-added 161Tb as an alternative to the clinically-applied 177Lu for radionuclide therapy
Source: EJNMMI Radiopharm Chem. 2019 Jul 10;4:12. doi: 10.1186/s41181-019-0063-6 (PMC6620226; doi:10.1186/s41181-019-0063-6)
Supplement: Supplementary file 1 — Table S1. Chemical admixtures of 160Gd2O3, provided by the supplier (Isoflex, USA). Table S2. Long-lived radioactive tracers used for bench experiments. Figure S1. Elution profile of Tb/Gd separation from the target material (10 mm x 170 mm Sykam resin column, 0.6 mL/min eluent flow rate). Experiment 1 (a) was run without addition of natGd2O3, while Experiment 2 (b) was performed with the addition of 140 mg natGd2O3. Figure S2. Elution profile of Tb separation from the target material (Gd) and the impurities of the target material (10 mm x 170 mm Sykam resin column, 0.6 mL/min eluent flow rate). Experiment 3 (a) 65Zn and 22Na were added to the system as impurities. Experiment 4 (b) 59Fe, 51Cr and 65Ni were added to the system as impurities. Figure S3. Elution profile of Tb separation from Cr as a possible radioactive impurity in the final 161Tb product (6 mm x 5mm LN3 resin column, 0.6 mL/min eluent flow rate). Figure S4. Gamma spectrum of the decayed product (161TbCl3) used for the determination of 160Tb radionuclidic impurity in the total 161Tb fraction. No other radionuclides, other than 160Tb were found. Figure S5. Radio TLC chromatogram of 161TbCl3 solution in 0.1 M sodium citrate (pH 5.5) for the determination of 161Tb radiochemical purity. (DOCX 911 kb) [file 41181_2019_63_MOESM1_ESM.docx]

Supplementary Material

**Production and characterization of no-carrier-added ^161^Tb as an alternative to the clinically-applied ^177^Lu for radionuclide therapy**

Nadezda Gracheva^1^, Cristina Müller^1^, Zeynep Talip^1^, Stephan Heinitz^2^, Ulli Köster^3^, Jan Rijn Zeevaart^4^, Alexander Vögele^2^, Roger Schibli^1,5^, Nicholas P. van der Meulen^1,2*^

*^1^ Center for Radiopharmaceutical Sciences ETH-PSI-USZ, Paul Scherrer Institut, 5232 Villigen-PSI, Switzerland*

*^2^ Laboratory of Radiochemistry, Paul Scherrer Institut, 5232 Villigen-PSI, Switzerland*

*^3^ Institut Laue-Langevin, 38042 Grenoble, France*

*^4^ Radiochemistry, South African Nuclear Energy Corporation (Necsa), 0242 Brits, South Africa*

*^5^ Department of Chemistry and Applied Biosciences, ETH Zurich, 8093 Zurich, Switzerland*

E-mail addresses:

[nadezda.gracheva@psi.ch](mailto:nadezda.gracheva@psi.ch); [cristina.mueller@psi.ch](mailto:cristina.mueller@psi.ch); [zeynep.talip@psi.ch](mailto:zeynep.talip@psi.ch); [stephan.heinitz@sckcen.be](mailto:stephan.heinitz@sckcen.be); [koester@ill.fr](mailto:koester@ill.fr); [janrijn.zeevaart@necsa.co.za](mailto:janrijn.zeevaart@necsa.co.za); [alexander.voegele@psi.ch](mailto:alexander.voegele@psi.ch); [roger.schibli@psi.ch](mailto:roger.schibli@psi.ch); [nick.vandermeulen@psi.ch](mailto:nick.vandermeulen@psi.ch)

***Corresponding author**:

Dr. Nicholas P. van der Meulen

Laboratory of Radiochemistry/Center for Radiopharmaceutical Sciences ETH/PSI/USZ

Paul Scherrer Institut

5232 Villigen-PSI

Switzerland

e-mail: nick.vandermeulen@psi.ch

phone: +41-56-310 50 87

fax: +41-56-310 28 49

**Development of the ^161^Tb purification process on the bench**

**Purpose:** After irradiation of ^160^Gd targets, a purification process of the radionuclide of interest is required. This step is critical towards obtaining radionuclidically, radiochemically and chemically pure ^161^Tb, relevant for further preclinical or clinical application. The purification method should be reproducible and suitable for the separation of µg amounts of the radionuclide (e.g. the mass of 20 GBq ^161^Tb is 4.6 µg) from mg amounts of the target material.

**Methods:** Bench experiments using a Sykam cation exchange resin column were performed with the use of radioactive tracers in order to establish experimental conditions for appropriate Tb separation from Gd target material and impurities related to the target material (Table S1).

**Table S1.** Chemical admixtures of ^160^Gd_2_O_3_, provided by the supplier (Isoflex, USA)

| **Element** | K | Na | Ca | Mg | Fe | Al | Si | Cr | Ni | Cu | Pb | Sb | Sn |
| --- | --- | --- | --- | --- | --- | --- | --- | --- | --- | --- | --- | --- | --- |
| **Content (ppm)** | <50 | <20 | <50 | <3 | <50 | <3 | <50 | <5 | <1 | <10 | 13 | <1 | <1 |

| **Element** | Pt | Sm | Ho | Dy | Eu | Nd | Tb | Er |
| --- | --- | --- | --- | --- | --- | --- | --- | --- |
| **Content (ppm)** | 20 | 13 | 6 | <1 | <1 | <1 | <2 | <1 |

Long-lived radioactive tracers **(**^22^Na, ^65^Zn, ^152^Eu, ^153^Gd, ^160^Tb, ^192^Ir) were provided by the Laboratory of Radiochemistry (PSI) (Table S2). ^59^Fe and ^51^Cr were obtained by neutron activation of the OPTIFER-V material (Saarschmiede GmbH, Germany) at SINQ (PSI). The required mass of the steel (383 mg) was placed inside the plastic capsule for irradiation and bombarded for 1 h at the NAA position of the SINQ spallation source. After the irradiation, the steel was dissolved in 2.0 mL 37% hydrochloric acid (HCl, Normapur, VWR Chemicals, USA) and evaporated until dryness at 80 °C under gas flow. ^65^Ni tracer was obtained by 1 h neutron irradiation of 12 mg nickel(II) nitrate hexahydrate (Ni(NO_3_)_2_.6H_2_O, Sigma-Aldrich, USA) at the NAA position (SINQ), followed by the same dissolving procedure as for ^59^Fe and ^51^Cr.

**Table S2.** Long-lived radioactive tracers used for bench experiments.

| **Tracer** | ^22^Na | ^51^Cr | ^59^Fe | ^65^Ni | ^65^Zn | ^152^Eu | ^153^Gd | ^160^Tb | ^169^Yb |
| --- | --- | --- | --- | --- | --- | --- | --- | --- | --- |
| **T_1/2_** | 2.6 y | 27.7 d | 44.5 d | 2.5 h | 244.3 d | 13.5 y | 239.5 d | 72.3 d | 32.0 d |

The radioactive tracers were mixed with 2.0 mL 0.1 M NH_4_NO_3_ and loaded onto the Sykam resin column (10 mm x 170 mm; NH_4_^+^ form) by means of an Ismatec peristaltic pump (Cole-Parmer Instrument Company LLC, USA). Subsequently, 0.13 M (pH 4.5) α-hydroxy-isobutyric acid (α-HIBA, Sigma-Aldrich GmbH, Germany) was passed through the column until Tb was eluted. The concentration of α-HIBA was then increased to 0.175 M and 1 M, respectively, in order to elute the impurities in question. Samples (6-12 mL fractions) of α-HIBA were collected and measured using a high-purity germanium (HPGe) detector (Canberra, France) in combination with the InterWinner software package (version 7.1, Itech Instruments, France) until the 3σ uncertainty was below 10%. In several experiments, ^153^Gd tracer was mixed with 8 – 140 mg ^nat^Gd_2_O_3_ (Research chemicals, Division of Rhone-Poulenc Inc., USA).

The Tb-containing α-HIBA fractions eluted from the Sykam resin were loaded onto the LN3 resin (LN3, Triskem International, France) column (6 mm x 5 mm) in order to concentrate Tb. After loading, the resin was rinsed with 10 mL MilliQ water, followed by the final elution of Tb in 0.05 M HCl.

**Results:** Initially, ^153^Gd and ^160^Tb tracers were used to establish conditions for appropriate Tb/Gd separation on the Sykam resin column with the use of α-HIBA as eluent (Experiment 1; Fig. S1a). The ^152^Eu, present in the experiment, was an impurity of the provided ^153^Gd solution; however, this allowed one to understand the behavior of Eu as a possible impurity of the target material for ^161^Tb production (Table S2). After the experimental conditions for appropriate Tb/Gd separation were determined (0.13 M α-HIBA, pH 4.5; 0.6 mL/min flow rate) with the corresponding tracers, similar conditions were applied for the Tb separation of up to 140 mg of ^nat^Gd_2_O_3_ (Experiment 2; Fig. S1b).


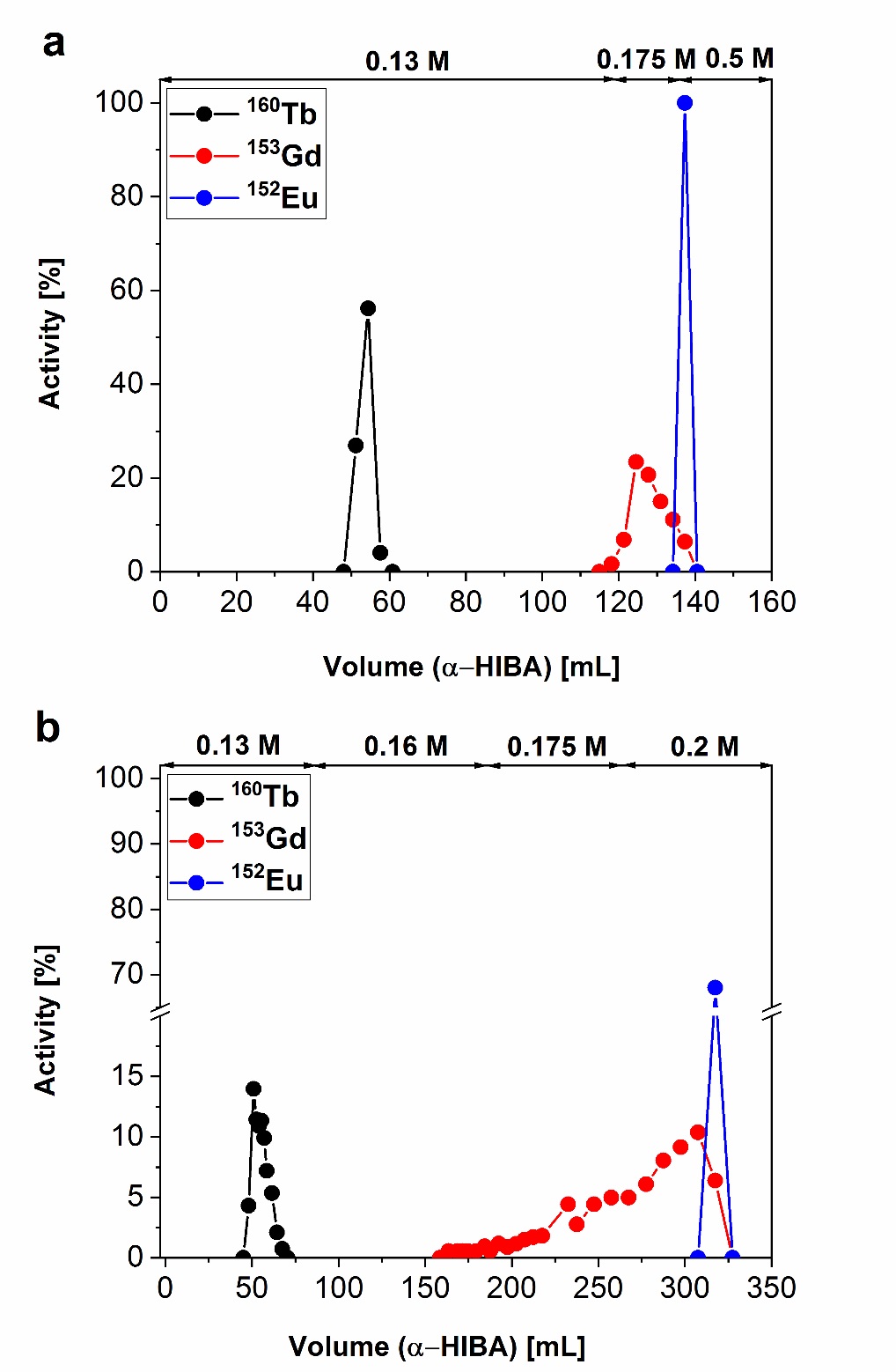


**Fig. S1.** Elution profile of Tb/Gd separation from the target material (10 mm x 170 mm Sykam resin column, 0.6 mL/min eluent flow rate). Experiment 1 (**a**) was run without addition of ^nat^Gd_2_O_3_, while Experiment 2 (**b**) was performed with the addition of 140 mg ^nat^Gd_2_O_3_.

Even in the presence of 140 mg of ^nat^Gd_2_O_3_ in the system (Experiment 2), Tb was effectively separated from ^nat/153^Gd (Fig. S1b). With the increase of the ^nat^Gd mass, a significant fronting of the Gd peak was observed due to the expanded longitudinal occupation of the column volume. This zone broadening had no effect with regard to overlap of the Tb peak, however.

The next step was to study the behavior of various radioactive tracers in order to simulate the presence of potential impurities (Table S2) in the ^161^Tb chemical separation system (Fig. S2a, b). The purpose of Experiment 3, shown in Fig. S2a, was to understand the behavior of Zn and Na (or K) in simulated Tb production conditions, while the purpose of Experiment 4 (Fig. S2b) was to evaluate the behavior of Fe, Cr and Ni on the Sykam resin column – also simulating Tb production conditions.

In Experiment 4, the concentration of α-HIBA was increased from 0.13 M to 0.175 M before Tb was eluted from the Sykam column, such that one could speed up the elution of ^65^Ni (its short 2.5 h half-life hinders γ-spectrometric ^65^Ni detection in more time-consuming experiments). This resulted in the overlapping of Tb and Gd peaks.

**
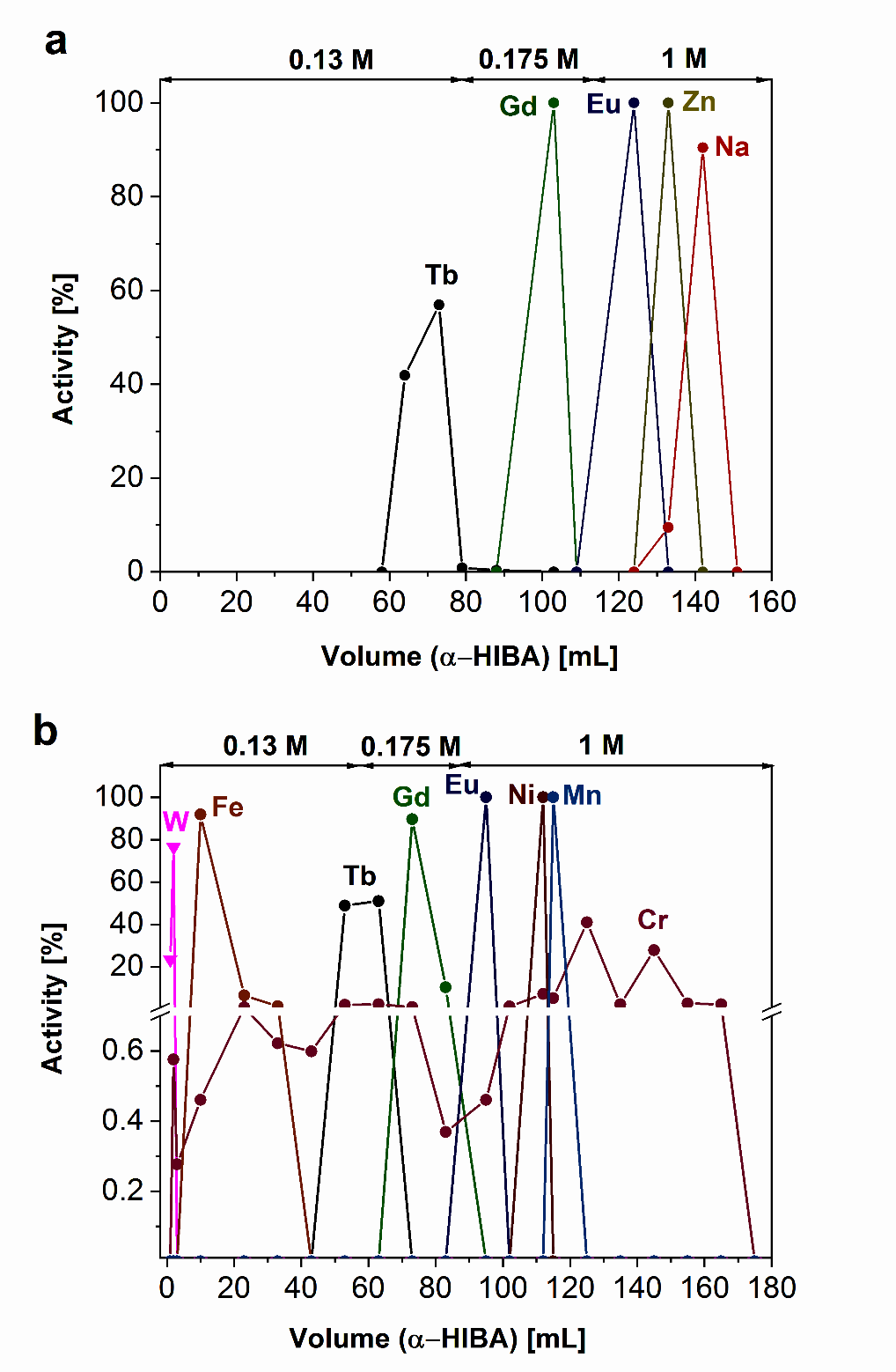
**

**Fig. S2.** Elution profile of Tb separation from the target material (Gd) and the impurities of the target material (10 mm x 170 mm Sykam resin column, 0.6 mL/min eluent flow rate). Experiment 3 (**a**) ^65^Zn and ^22^Na were added to the system as impurities. Experiment 4 (**b**) ^59^Fe, ^51^Cr and ^65^Ni were added to the system as impurities.

The separation of Tb from the possible impurities of the target material on the Sykam resin column showed that Cr species were eluted at different concentrations of α-HIBA and resulted in the presence of Cr in Tb fraction (Experiment 4; Fig. S2b). Cr ions form oligomers (monomers to tetramers) in aqueous solution, which may interact individually with the eluent [1]. Unfortunately, the complexation mechanisms and complexation constants of such Cr species with α-HIBA are not known and it is difficult to explain why traces of Cr were eluted in every fraction throughout the separation process. Nevertheless, Cr was not retained on the LN3 resin column (Column 2 of the purification process, 6 mm x 5 mm), which allowed efficient Tb separation from Cr as an impurity in the ^161^Tb final product (Fig. S3).

**

**

**Fig. S3.** Elution profile of Tb separation from Cr as a possible radioactive impurity in the final ^161^Tb product (6 mm x 5 mm LN3 resin column, 0.6 mL/min eluent flow rate).

**Conclusion:** Experimental conditions for appropriate Tb separation from the Gd target material and potential impurities were established by performing bench experiments. These conditions were successfully translated for the purification of the reactor-produced ^161^Tb on the constructed purification module (discussed in the main manuscript).

**^161^Tb radionuclidic purity**

The only radionuclidic impurity, found in the decayed ^161^Tb product, was ^160^Tb (T_1/2_ = 72.3 d) which was <0.007% of the total ^161^Tb activity at the end of separation (EOS) (Fig. S4). The absence of the long-lived ^153^Gd (T_1/2_ = 239.5 d) in the decayed product, produced via the ^152^Gd(n,γ)^153^Gd nuclear reaction, emphasizes the efficient ^161^Tb separation from the Gd target material.

**
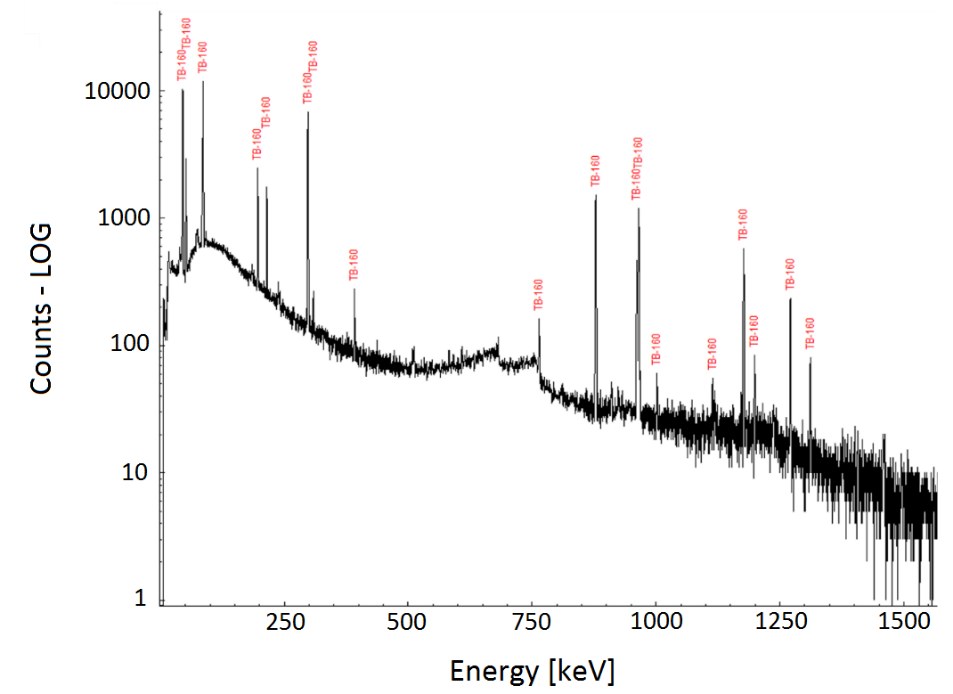
**

**Fig. S4.** Gamma spectrum of the decayed product (^161^TbCl_3_), used for the determination of ^160^Tb radionuclidic impurity in the total ^161^Tb fraction. No other radionuclides, other than ^160^Tb, were found.

**^161^Tb radiochemical purity**

The radiochemical purity of ^161^Tb solution was determined by radio thin layer chromatography (radio TLC). Each batch of ^161^Tb had >99% radiochemical purity. The absence of the *R*_f_ 0 fraction (which would indicate Tb species not forming complexes with citrate, e.g. colloids) on the radio TLC chromatogram (Fig. S5) indicates the existence of only ^161^Tb^3+^ in the final product.

**
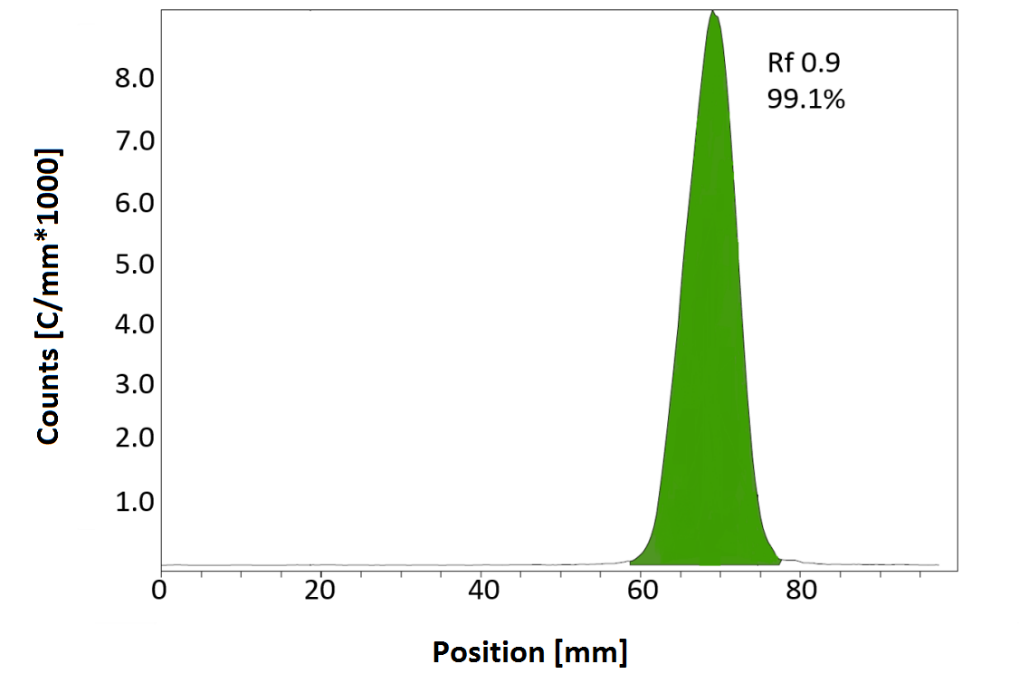
**

**Fig. S5.** Radio TLC chromatogram of ^161^TbCl_3_ solution in 0.1 M sodium citrate (pH 5.5) for the determination of ^161^Tb radiochemical purity.

**References**

[1] Friese JI. Chromatographic separation and characterization of hydrolyzed Cr(III) species*.* Anal. Chem. 2002;74:2977-84.
